# Effect of Subsoiling on the Nutritional Quality of Grains of Maize Hybrids of Different Eras

**DOI:** 10.3390/plants13141900

**Published:** 2024-07-10

**Authors:** Liqing Wang, Xiaofang Yu, Julin Gao, Daling Ma, Tong He, Shuping Hu

**Affiliations:** College of Agronomy, Inner Mongolia Agricultural University, Hohhot 010019, China; nmwangliqing@emails.imau.edu.cn (L.W.); madaling@sina.com (D.M.); hetong0124@163.com (T.H.); bthsp88@163.com (S.H.)

**Keywords:** spring maize, subsoiling, grain nutritional quality

## Abstract

To achieve high maize (*Zea mays* L.) yields and quality grain, it is necessary to develop stress-resistant cultivars and related cultivation practices, aiming to maximize efficiency. Thus, our objectives were (i) to investigate the impact of tillage practices and maize hybrids (which have improved over time) on yield and its components, and (ii) to characterize the response pattern of maize hybrid grain nutrient quality components to subsoiling. To achieve this, we conducted field trials with five maize hybrids from different eras under two tillage practices: rotary tillage and subsoiling. We compared grain yield, nutritional quality, and other indicators across different tillage conditions from the 1970s to the 2010s. The main results of this study are as follows: under rotary tillage conditions, the 2010s hybrid (DH618) significantly increased yields (9.37–55.89%) compared to hybrids from the 1970s–2000s. After subsoiling, the physiologically mature grains of all hybrids exhibited minimal changes in crude protein and fat content, while there was a significant reduction in the total soluble sugar content of the grains. After subsoiling, there was a substantial 8.14 to 12.79 percent increase in total starch accumulation in the grain for all hybrids during the period of 47–75 days post-anthesis. Furthermore, during the period of 47–75 days after anthesis, the consumption of grain crude protein significantly contributed to the accumulation of total starch in the grains. Ultimately, subsoiling significantly increased the yield of each hybrid and enhanced the total grain starch content at physiological maturity of all hybrids, with the 2010s hybrid (DH618) performing exceptionally well.

## 1. Introduction

Soil health is essential for improving the quality of arable land and achieving sustainable agricultural development. The physical, chemical, and biological properties of soil are closely related to soil health [1,2]. However, modern agriculture’s intensive land use to maximize crop yields poses a serious threat to soil health and food security [3]. Maize (*Zea mays* L.) is a significant food crop and plays a crucial role in human diets. Therefore, there is a need to focus on enhancing the nutritional quality of maize grains while ensuring high yields, especially with the ongoing agricultural and rural economic transformations [4]. Inner Mongolia, as China’s main grain production area, has long relied on traditional tillage practices that result in shallow plough layers, decreased nutrient utilization, and persistent soil issues. These factors significantly impact maize growth and compromise the nutritional quality of maize grains [5].

Tillage methods are crucial for enhancing the quality of the plow layer and improving soil fertility, thus playing a significant role in both soil health and crop nutritional quality [6,7,8]. Currently, China employs various plowing methods for farmland, including traditional plowing (shallow and rotary plowing), deep plowing (subsoiling and deep tilling), and no-tillage (stubble mulching). These different plowing methods have distinct impacts on soil properties such as bulk weight, porosity, water content, pH, carbon, nitrogen, nutrient content, and soil microorganisms [9,10]. Conservation tillage practices, including no-tillage and minimal tillage, have the potential to lower wind and water erosion, as well as decrease water evaporation from the field, which has the effect of changing soil fertility and saving costs and increasing efficiency [11,12]. Nevertheless, no-tillage can lead to an increase in soil bulk density and compaction [13], diminish soil temperature [14], and impede maize root growth, thereby hindering nutrient absorption from deeper soil strata. Subsoiling, a common agricultural practice, effectively loosens the soil, increases soil organic carbon (SOC) content in the 0–50 cm soil layer, and breaks up the plow subsoil [15,16]. Moreover, subsoiling reduces surface runoff, improves water utilization, and thereby promotes crop growth, enhances crop yields, and contributes to improve the potential for sustainable development of farmland [17,18]. Additionally, it has been observed that subsoiling has positively affected various yield components while increasing maize yield [19].

Maize grains are abundant in nutrients including starch, protein, fat, water-soluble polysaccharides, vitamins, and minerals [20]. Among these components, starch, as a sustainable and cost-effective biodegradable natural polysaccharide material, comprises approximately 65–70% of the entire endosperm [21]. Proteins are essential components of living organisms, playing crucial roles in energy metabolism. At physiological maturity, storage proteins in maize kernels constitute approximately 10% of the grain weight. Maize grains have a crude fat content of approximately 4–6%, making them a viable raw material for the production of edible oils [22]. The yield and nutritional quality of maize are determined during the process of photosynthetic product accumulation and distribution [23]. Consequently, many cultivation and management practices that substantially impact maize yield also have a significant influence on the nutritional quality of maize grains.

The process of grain filling plays a crucial role in determining the weight of maize grains, and the formation of nutritional quality is primarily concentrated during the stage of material accumulation in the yield organs. Logistic equations are frequently employed to accurately describe the grain filling process and calculate essential grain filling parameters [24]. Results from studies on cereal crops have demonstrated that tillage practices have a significant impact on several vital grain filling parameters, including the average grouting rate (Gave), maximum grouting rate (Gmax), grain weight at the time of attaining the maximum grouting rate (Wmax), time at maximum grain filling (Tmax), and the active grouting period (AGP), subsequently influencing grain weight [25,26]. Additionally, our previous research found that the effects of subsoiling on grain filling rate and duration were distinct across different filling stages. In the rapid and slow growth stages, subsoiling increased the average filling rate for the entire filling stage by enhancing grain filling rate. Conversely, in the gradual growth stage, subsoiling extended the active filling period for the entire filling stage by prolonging grain filling duration [27].

Similar to most cereal crops, the nutritional composition of maize grains primarily consists of starch, crude protein, and crude fat. At physiological maturity, maize grains typically contain approximately 70% starch, 8–11% crude protein, and 4–6% crude fat [28,29]. Nutrient accumulation within cereal grain is not uniform. During normal maturation conditions, protein synthesis predominantly occurs in the early stages of filling, while starch synthesis is less active at this stage. From the beginning of milk ripening to wax ripening, sugars are transported more efficiently to the grain, resulting in greater starch synthesis compared to protein synthesis. In the later stages of grain development, sugar transport to the grain weakens or halts, while nitrogen inputs persist [30,31]. Furthermore, the effects of subsoiling tillage on the nutrient quality components of maize grains at different stages of grain filling vary, as highlighted by previous studies. Therefore, the objectives of this study were twofold: (i) to investigate the impact of tillage practices and maize hybrids (which have improved over time) on yield and its components, and (ii) to characterize the response pattern of maize hybrid grain nutrient quality components to subsoiling.

## 2. Results

### 2.1. Effect of Subsoiling on Yield and Its Components in Maize Hybrids of Different Eras

**Figure 1:**
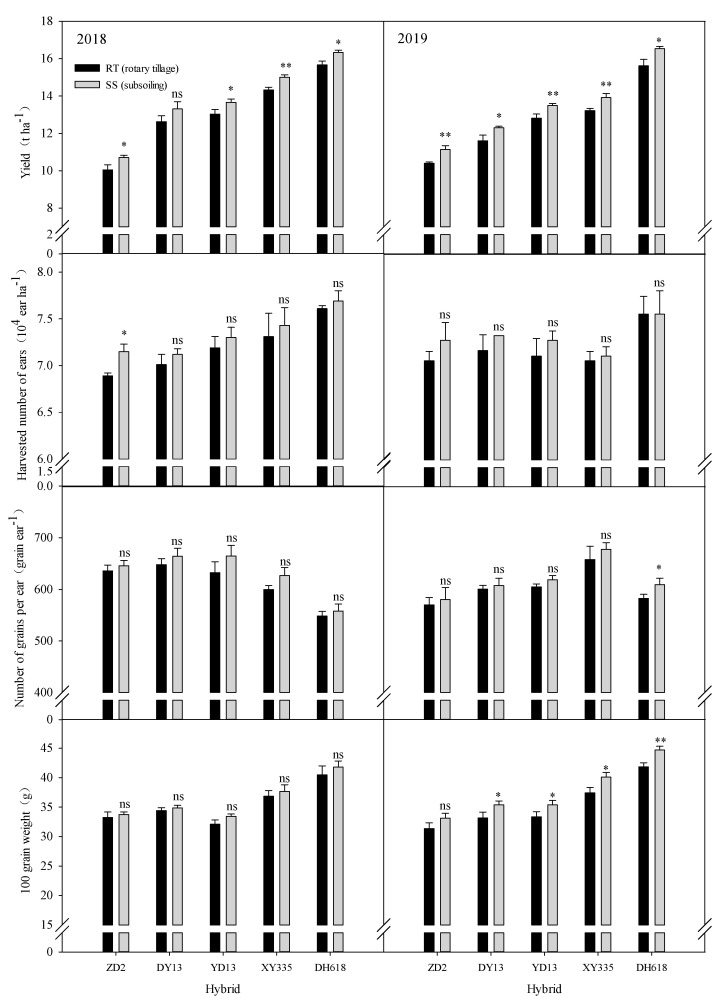
Effects of subsoiling on the yield and its components of different eras’ maize hybrids. “ns,” “*”, and “**” are used to demonstrate differences between tillage practices. Numbers indicate the magnitude of change in indicators between tillage methods. “ns”: not significant at *p* < 0.05. “*”: significant at *p* < 0.05; “**”: significant at *p* < 0.01.

Figure 1 illustrates that the yield, harvested number of ears, and 100-grain weight of the 2010s hybrid (DH618) were significantly greater than the hybrids from other eras. However, the 2010s hybrid had a significantly lower number of grains per ear compared to the hybrids from other eras. Subsoiling had a significant impact on the yield of maize hybrids from different eras, with the highest increase observed in the 2010s hybrids: 0.67 t·ha^−1^ (2018) and 0.92 t·ha^−1^ (2019). Regarding yield components, the harvested number of ears, the number of grains per ear, and the 100-grain weight of each hybrid of the 1970s to 2010s exhibited varying changes following subsoiling. In 2018, the only significant difference observed between the tillage methods was in the harvested number of ears of the 1970s hybrid (ZD2). In 2019, significant differences were found between the tillage methods in terms of the number of grains per ear for the 2010s hybrid. Additionally, the 100-grain weight exhibited significant differences among the 1980s–2010s hybrids and showed the highest increase of 2.86 g. The data suggest that the yield increase in 2019 was more pronounced with subsoiling, with the 2010s hybrid (DH618) displaying a higher potential for yield improvement. This potential can be attributed primarily to the higher number of grains per ear and 100-grain weight, both of which were enhanced by subsoiling.

### 2.2. Effect of Subsoiling on Grain Crude Protein Content in Maize Hybrids of Different Eras

**Figure 2:**
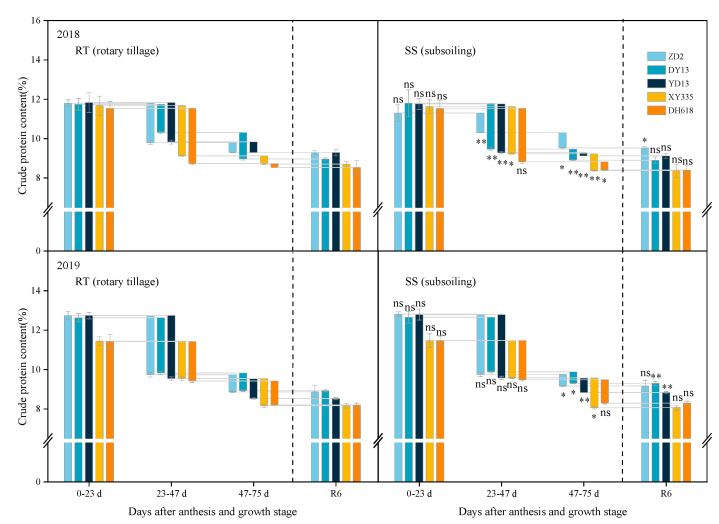
Effects of subsoiling on the crude protein content of grains of different eras’ maize hybrids. “ns,” “*”, and “**” are used to demonstrate differences between tillage practices. “ns”: not significant at *p* < 0.05. “*”: significant at *p* < 0.05; “**”: significant at *p* < 0.01.

The 2010s hybrid (DH618) exhibited significantly lower grain crude protein content at physiological maturity compared to the 1970s–1990s hybrids under rotary tillage conditions. In contrast to rotary tillage, the variations in the grain crude protein content of hybrids of different eras in response to subsoiling varied significantly across various growth stages (Figure 2). After anthesis, between 47 and 75 days, in 2018, the reduction in grain crude protein content displayed a significant decline of 57.83% and 69.19% in hybrids from the 1980s and 1990s (DY13 and YD13), respectively, whereas hybrids from the 1970s, 2000s, and 2010s (ZD2, XY335 and DH618) exhibited a significant increase of 54.13%, 105.08%, and 131.32% in the same period. Under consistent conditions in 2019, there was a notable increase of 9.90% in the crude protein content reduction of grains from hybrids of the 2000s decade. Conversely, hybrids from the 1970s, 1980s, 1990s, and 2010s exhibited significant decreases of 32.52%, 35.04%, 25.96%, and 2.41% respectively. Also at physiological maturity, a noteworthy increase of 2.51% in grain crude protein content was noted for hybrids from the 1970s in 2018, and 4.10% and 3.52% for hybrids from the 1980s and 1990s in 2019.

### 2.3. Effect of Subsoiling on Grain Total Starch Content in Maize Hybrids of Different Eras

**Figure 3:**
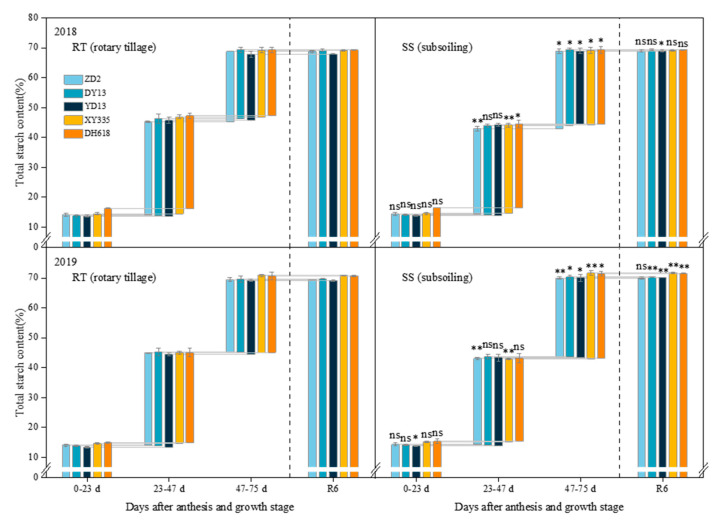
Effects of subsoiling on the total starch content of grains of different eras’ maize hybrids. “ns,” “*”, and “**” are used to demonstrate differences between tillage practices. “ns”: not significant at *p* < 0.05. “*”: significant at *p* < 0.05; “**”: significant at *p* < 0.01.

In contrast to rotary tillage, the variations in the grain total starch content of hybrids of different eras in response to subsoiling varied significantly across various growth stages (Figure 3). Following subsoiling, the incremental total starch content of the grains was significantly increased by 9.86%, 9.99%, 11.37%, 12.49%, and 12.79% during the period of 47–75 days after anthesis in 1970s–2010s hybrids in 2018. Under identical conditions in 2019, the incremental total starch content in grains of hybrids spanning from the 1970s to the 2010s showed a significant increase of 9.56%, 8.77%, 8.14%, 11.63%, and 10.26%. Also at physiological maturity, in 2018, only the grains of the 1990s hybrid showed a significant increase of 1.52% in total starch content; in 2019, the grains of hybrids from the 1980s to the 2010s exhibited significant increases of 0.91%, 1.25%, 1.18%, and 1.13% in total starch content. The impact of subsoiling on the total starch content of grains at physiological maturity is evident through the enhanced accumulation of starch during the period of 47–75 days post-anthesis. Notably, the increase in total starch content in grains of the 2000s–2010s hybrids during this period showed a more pronounced response to subsoiling.

### 2.4. Effect of Subsoiling on Grain Crude Fat Content in Maize Hybrids of Different Eras

**Figure 4:**
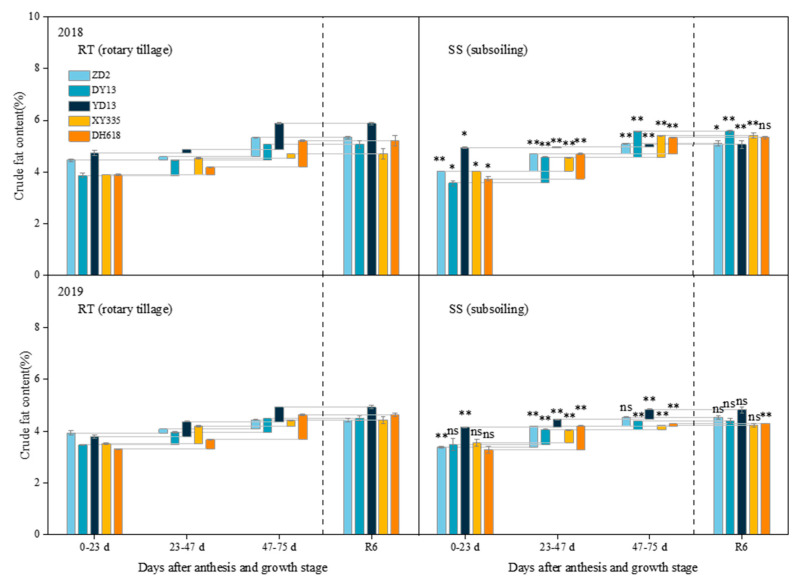
Effects of subsoiling on the crude fat content of grains of different eras’ maize hybrids. “ns,” “*”, and “**” are used to demonstrate differences between tillage practices. “ns”: not significant at *p* < 0.05. “*”: significant at *p* < 0.05; “**”: significant at *p* < 0.01.

In contrast to rotary tillage, the variations in the grain crude fat content of hybrids of different eras in response to subsoiling varied significantly across various growth stages (Figure 4). Following subsoiling, the most significant variation in the increase in grain crude fat content was noted in the 1970s hybrid during the period of 23–47 days after anthesis, reaching 394.05% in 2018 and 406.66% in 2019 across the two growing seasons. During the period of 47–75 days after anthesis, the highest rise in incremental grain crude fat content (395.03%) occurred in the 2000s hybrid in 2018, while the most significant decrease (−90.42%) was witnessed in the 2010s variety in 2019, all under subsoiling conditions. Also at physiological maturity, the hybrid from the 1990s exhibited the most significant reduction in grain crude fat content in 2018, with a decrease of 13.64%, while the hybrid from the 2010s recorded a reduction of 7.47% in 2019.

### 2.5. Effect of Subsoiling on Grain Total Soluble Sugar Content in Maize Hybrids of Different Eras

**Figure 5:**
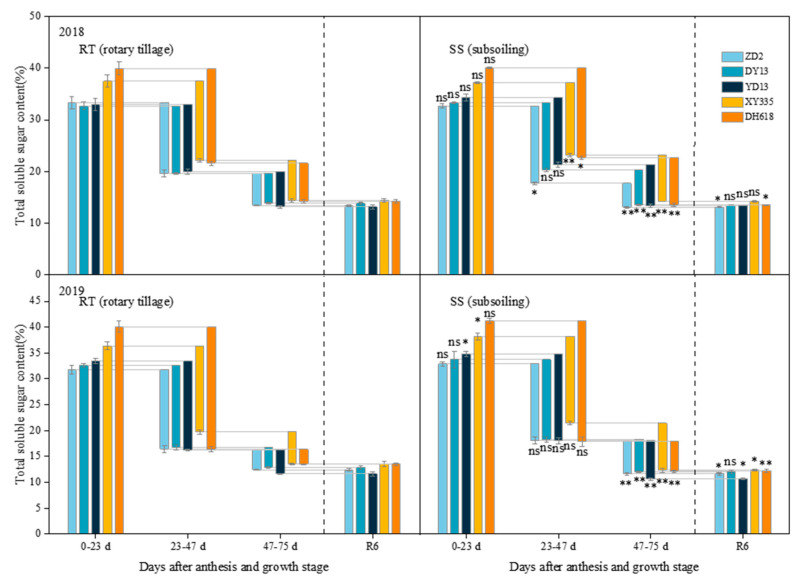
Effects of subsoiling on the total soluble sugar content of grains of different eras’ maize hybrids. “ns,” “*”, and “**” are used to demonstrate differences between tillage practices. “ns”: not significant at *p* < 0.05. “*”: significant at *p* < 0.05; “**”: significant at *p* < 0.01.

The total soluble sugar content of the grains at physiological maturity was notably higher in the hybrid from the 2010s compared to those from the 1970s and 1990s in both growing seasons under rotary tillage (Figure 5). In contrast to rotary tillage, the variations in grain crude fat content of hybrids of different eras in response to subsoiling varied significantly across various growth stages. After subsoiling, the decrease in the total soluble sugar content of the grains of 1980s–2010s hybrids further increased during the period of 47–75 days after anthesis, with the greatest increase in the decrease in total soluble sugar content of the grain of 2010s hybrid, which was 20.29% (2018) and 96.27% in the two growing seasons, respectively. The hybrid from the 2010s also exhibited the most significant decline in total soluble sugar content of the grains at physiological maturity, with reductions of 5.25% (2018) and 9.84% (2019) in the two growing seasons.

### 2.6. Relationship between Yield and Nutritional Quality of Grains at Physiological Maturity

**Figure 6:**
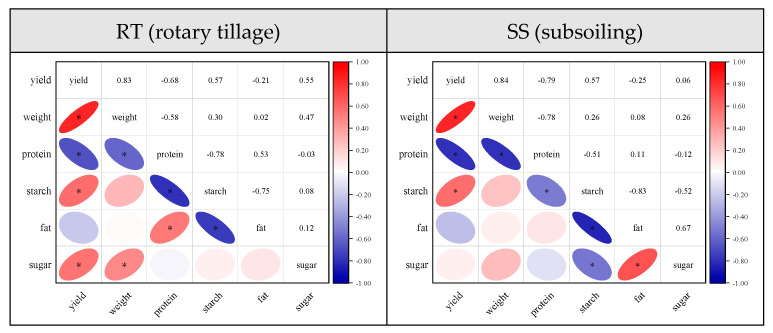
Correlation analysis of yield and grain nutrient quality. “*” indicates significant differences at *p* < 0.05.

The findings of the correlation analysis indicated that higher total starch content and lower crude protein content in grains at physiological maturity were associated with increased maize yield and higher 100-grain weight (Figure 6). Simultaneously, the association between the total soluble sugar content of maize kernels at physiological maturity and the 100-kernel weight and yield of maize exhibited variations across distinct tillage practices. Total soluble sugar content of grains was significantly and negatively correlated with yield and 100-grain weight under rotary tillage, whereas the correlation did not reach a significant level under subsoiling.

### 2.7. Interrelationships among the Components of Nutritional Quality of the Grains

A path analysis and stepwise regression analysis were performed to investigate the relationship between total starch accumulation in the grains at 47–75 days after anthesis and changes in crude protein content, crude fat content, and total soluble sugar content at three stages: 0–23 days after anthesis, 23–47 days after anthesis, and 47–75 days after anthesis. Additionally, the total starch accumulation in the grains at 0–23 days after anthesis and 23–47 days after anthesis was considered. Initially, X1, X2, X5, X6, X7, X8, X9, X10, and X11 were included in the regression equation through stepwise regression analysis. The parameters of the regression equation series can be found in Table 1.

The results of the pathway analysis revealed a significant positive correlation between the accumulation of total starch in grains from 47 to 75 days after anthesis and the variation in the content of crude protein in grains during the same period (Table 2). Additionally, there was a significant negative correlation between the accumulation of total starch in grains from 23 to 47 days after anthesis and the accumulation of total starch in grains from 47 to 75 days after anthesis. Furthermore, the variation in crude protein content in grains from 47 to 75 days after anthesis exhibited a positive correlation with the accumulation of total starch in grains during the same period, primarily through indirect effects. On the other hand, the variation in total starch content of grains during the period of 23–47 days after anthesis showed a negative correlation with the accumulation of total starch in grains from 47 to 75 days after anthesis, primarily through direct effects.

## 3. Discussion

Yield formation mirrors soil productivity, with past studies indicating improved crop yield formation in soils possessing advantageous physico-chemical properties [32]. Tillage exerts a critical influence on crop yield [33], and strategizing the tillage mode in tandem with fitting crop varieties constitutes an efficacious approach for maximizing crop yield [34]. In our research, an appreciable upswing in maize yield was noticed following the introduction of subsoiling plowing, corroborating findings by Li et al. [35]. Jug et al. [36] postulated that yield augmentation deriving from subsoiling could be ascribed to factors such as diminished soil infiltration resistance, elevated soil oxygen concentration, ameliorated soil moisture, and enhanced root growth. The interaction between plant access to water and nutrient effects was also noted. Maize yield hinges on population size and individual yield, with the weight of a thousand grains decreasing as grains per ear increase, given a constant count of ears per unit area [37]. In comparing subsoiling to rotary rotation, divergent views exist. Some posit that subsoiling can inflate the count of ears and grains in each ear, and the weight of a thousand grains [38], while others suggest it is more likely to augment maize plant productivity with a negligible effect on the abundance of effective ears [39]. These inconsistent results could stem from variations in cropping systems and fertility and cropping density conditions under which past studies have been conducted. In our study, yield per unit area rose primarily due to increased yield per individual plant—specifically in terms of the number of ears and the 100-grain weight—with the 2010s hybrid (DH618) showing more responsiveness to subsoiling. The effect of subsoiling on effective ear count was insignificant in our study, potentially due to our use of a lower planting density. Suitable tillage practices can bolster soil physico-chemical properties, thereby enhancing grain yield in intensive maize production systems. Under prevailing intensive maize cultivation conditions, many studies recommend employing subsoiling practices to counteract prolonged monoculture tillage restrictions [40,41].

Tillage practices profoundly alter the nutrient profile of cereal crops by reshaping the micro-ecological balance involving soil water, fertilizer, air, heat, and pivotal enzymes engaged in grain component synthesis and transformation [42]. This study found that subsoiling augmented the total grain starch content at physiological maturity as compared to rotary tillage, while exhibiting only a minor impact on the crude protein content. This could be due to subsoiling’s enhancement of soil moisture and its various physiological and ecological benefits, such as lowering water shortages during the filling period, subsequently resulting in tinier maize kernel starch granules and a prolonged average branching length of branched starch [43,44]. Additionally, greater fertilizer inputs corresponded to increased crude protein and fat content in maize grains during the pre-filling period, with a decrease in these components during the late filling period [45]. However, the effects of tillage on the examined traits were generally less significant compared to environmental factors, variety, and input level, becoming conspicuous only in combination with specific input levels. This study supports these findings, with total soluble sugar levels in grains at physiological maturity under rotary tillage fluctuating considerably among varieties (9.34–16.43%), and being less variable under tillage methodologies (1.17–9.84%) [46]. Yield formation is fundamentally based on carbohydrate accumulation and conversion by the grain, with grain filling characteristics reflecting this physiological process [47]. It is well known that the increased ratio of the aleurone layer fraction to whole grains is often caused by poorly filling grains with lower starch grains, consequently leading to the higher values of measured protein and fat content (%) [48,49]. In our study, the YD13 hybrid from the 1990s demonstrated lower total starch content in the grain at physiological maturity, in line with our earlier findings of a significantly lower grouting rate for this hybrid [27]. Both tillage practices and variety interactions markedly influenced the total starch content in maize grains. Nevertheless, the rise in total starch content following subsoiling differed among varieties, with the ZD2 hybrid from the 1970s recording the smallest increase (0.51%) [50]. This discrepancy might be ascribed to variations in post-flowering growth stage duration, as prior research indicates that subsoiling tillage can retard leaf senescence during plants’ late reproductive stage, thereby enhancing post-flowering dry matter grain accumulation [51]. The delay between male pumping and expelling in older hybrids is longer, leading to rapid leaf senescence and diminished dry matter accumulation post-flowering. As such, the increase in total starch content from the interplay between older hybrids and subsoiling tillage was also less substantial [52].

**Figure 7:**
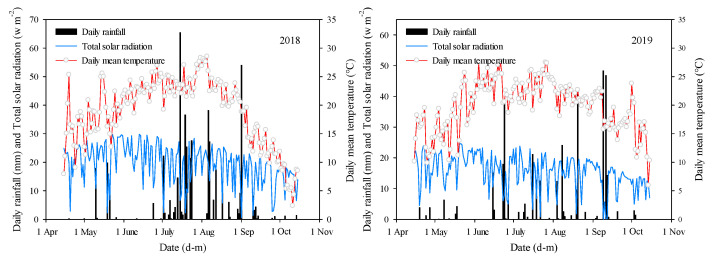
Main meteorological factors during the growth period in the experimental area.

The soil carbon-to-nitrogen (C/N) ratio serves as a pivotal metric in determining soil quality by showcasing its capacity to store and recycle energy alongside nutrients [53]. High-C/N ratio soils are often associated with rapid nitrogen fixation, whereas those with low C/N ratios exhibit slower processes [54]. Greater carbon retention in soils is a product of conservation tillage practices, as opposed to conventional tillage. This results in better soil structural conditions as well as improved nutrient conservation [55]. The association of higher soil C/N ratios with conservation tillage also affects the grain C/N ratios, illustrated by the note-worthy correlation between quantity yielded and grain’s total starch content (Figure 7). Higher plants primarily assimilate inorganic nitrogen through the GS/GOGAT cycle, and its subsequent conversion into a variety of amino acids via enzymes, such as GOT and GPT, promotes protein synthesis in grains [56,57]. This study found no significant impact of subsoiling on the grains’ crude protein content within 0–47 days post-anthesis. Starch, comprising straight-chain and branched-chain amylopectin, owes its biosynthesis in plant endosperm to a succession of synergistic enzymes [58]. AGP, for example, synthesizes adenosine diphosphate glucose, which is the primary substrate for starch synthesis [59]. One such enzyme, AGP, synthesizes adenosine diphosphate glucose, the primary substrate for starch synthesis [59]. The study revealed that subsoiling increased total grain starch accumulation for all hybrids within 47–75 days post-anthesis to a larger degree. However, total starch accumulation was reduced during the period of 23–47 days after anthesis. The new hybrid (DH618) exhibited the largest total starch content gain of 11.53% in the grain during the period of 47–75 days post-anthesis, attributed to its superior post-anthesis material production capability [60]. The existing literature suggests that stored proteins can fill gaps between starch grains, decrease voids, and facilitate a densely arranged starch structure [61]. Both starch and protein contents are integral to grain weight formation. Carbon metabolism provides the carbon skeleton and energy required for nitrogen metabolism, while nitrogen metabolism delivers key enzymes for carbon metabolism [62]. Therefore, subsoiling plays a coordinating role in carbon and nitrogen metabolism during the grain filling stage, modulating protein and starch synthesis throughout various filling stages. Nonetheless, the underlying physiological and molecular mechanisms warrant further investigation.

## 4. Materials and Methods

### 4.1. Description of Research Location

Field experiments were conducted at the Tumoteyou Qi Experimental Station of Inner Mongolia Agricultural University (40°33′ N, 110°31′ E) in 2018 and 2019. The soil at the experimental site is loam, with a 0–30 cm soil layer containing 22.27 g·kg^−1^ organic matter, 103.75 mg·kg^−1^ available nitrogen, 15.76 mg·kg^−1^ available phosphorus, and 219.60 mg·kg^−1^ available potassium, with a pH of 8.23. The main meteorological factors influencing maize growth during the study period are depicted in Figure 7.

### 4.2. Experimental Design

A farmland tilled by rotary tillage since 2014 was chosen. The farmland was divided into 2 plots. One plot was randomly selected and tilled by subsoiling followed with rotary tillage before maize sowing in 2018. The other plot was only subject to rotary tillage. In 2019, the same tillage practices as in 2018 were repeated on the same plot. The experiment was arranged in a split-zone plot design with two factors: tillage treatment and hybrid. The main plot consisted of two tillage treatments, namely subsoiling (SS) and rotary tillage (RT). Subsoiling depth was 35 cm, carried out with a vibration deep loosening machine, and spacing between two adjacent shovels was 35 cm.

Subsoiling was performed using a subsoiling plow (John Deere, IL, USA) attached to a John Deere 1654 tractor (John Deere, IL, USA). Rotary tillage depth was around 15 cm (Xinjiang Machinery Research Institute Co., Ltd., Xinjiang, China). The subplots included five maize hybrids: Zhongdan2 (ZD2, 1970s), Danyu13 (DY13, 1980s), Yedan13 (YD13, 1990s), Xianyu335 (XY335, 2000s), and Denghai618 (DH618, 2010s). The maximum annual acreage of each of these hybrids exceeds 400,000 ha. These hybrids are commercially available in Chinese markets and were purchased for testing purposes. Table 3 provides the release dates of the cultivars and parental lines of the hybrids. Each subplot was replicated three times. A planting density of 75,000 plants·ha^−1^ and a row spacing of 0.6 m were used. The plot area measured 6 m × 6 m. Basal fertilization included the application of ammonium phosphate dibasic and potassium sulfate before seeding. Ammonium dihydrogen phosphate (N 18%; P_2_O_5_ 46%) was applied at a rate of 375 kg·ha^−1^, and potassium sulfate (K_2_O 51%) was applied at a rate of 150 kg·ha^−1^. Urea (N, 46%) was utilized as a supplementary fertilizer with application rates of 30% at V6 (sixth leaf), 60% at V12 (12th leaf), and 10% at R2 (blister), with an overall rate of 345 kg·ha^−1^. Annually, during the entire growing cycle of maize, the following fertilizer quantities were utilized: 226.2 kg·ha^−1^ of pure nitrogen (N), 76.5 kg·ha^−1^ of potassium oxide (K_2_O), and 172.5 kg·ha^−1^ of phosphorus pentoxide (P_2_O_5_). Drip irrigation was administered four times throughout the growth stages: at V6, V12, R1 (silking), and R2. Each irrigation event encompassed an area of 750 m^3^·ha^−1^. All other management practices followed the standard procedures employed in large-scale farming operations.

### 4.3. Measurement

Grain Nutritional Quality: At three different time points (23 days, 47 days, and 75 days after anthesis), a representative cob was selected and the middle grains of the cob were placed in an oven at 105 °C for 30 min. They were then dried at 60 °C until reaching a constant weight before being crushed for measurement. The whole nitrogen content of the grains was determined using the semi-micro Kjeldahl method, and the crude protein content of the grains was calculated by multiplying the whole nitrogen content by 6.25 [23]. The crude fat content was determined using the Soxhlet extraction residue method [23], while the total starch and total soluble sugar content were determined using Yu et al.’s method [28].

Yield and Yield Components: During harvest, a 2 m by 5 m area in the center of each plot (consisting of two rows) was manually harvested, and the grains were dried to determine yield with 14% water content. Harvested ears were counted to determine the harvest ear density. Additionally, twenty ears were randomly selected, weighed, and used to measure the number of kernels per ear and the weight of 100 kernels.

### 4.4. Statistical Analysis

All the data were collected using Microsoft Excel 2019 (Microsoft, Inc., Redmond, WA, USA). The data were analyzed using SAS 9.4 (SAS Institute Inc., Raleigh, NC, USA) for *t*-test analysis, path analysis, stepwise regression, and correlation analysis. An independent-sample *t*-test was conducted to compare the yield, yield components, and nutritional quality components of the grains under different tillage methods each year during the same growth stage. Sigmaplot 12.5 (Systat Software, Inc., San Jose, CA, USA) was used to create the figures.

## 5. Conclusions

Under rotatory tillage conditions, the 2010s hybrid (DH618) displayed a significant increase in yield compared to hybrids from the 1970s to 2000s periods. This variation also occurs to different degrees within the various components of the grain’s nutritional quality at physiological maturation stage. The application of subsoiling techniques has notably enhanced the yield for all maize hybrids, while simultaneously influencing the content of the grain’s nutritional components at its maturity stage. The observed increase in the grain’s total starch content at physiological maturity can primarily be attributed to a substantial surge in grain starch accumulation during the period of 47–75 days following anthesis. Noteworthy is the intricate relationship between the nutritional quality components throughout the various growth stages. Particularly, during the 47–75 day period succeeding anthesis, a considerable reduction in grain crude protein was found to significantly boost the accumulation of total starch in the grain. Subsoiling contributed to the increase in total grain amylose content at physiological maturity by adjusting grain crude protein consumption and total grain amylose accumulation 47–75 days after anthesis, and during this process, the 2010s hybrid showed higher yield and total grain amylose content enhancement potential. Therefore, in cultivation and production processes where there are no special requirements for the nutritional quality components of maize grains, we recommend the use of the 2010s hybrid and the application of cultivation techniques with subsoiling, which can significantly increase maize yields by increasing the total starch content of the grains at physiological maturity.

## Figures and Tables

**Table 1:** Stepwise regression analysis of nutritional quality components of grains.

Index	B	Standard Error	F-Value	Sig.
Intercept	93.32	9.31	100.40	0.00
X1	−1.41	0.32	19.89	0.00
X2	−1.17	0.13	87.77	0.00
X5	0.79	0.31	6.41	0.03
X6	−2.62	0.86	9.28	0.01
X7	−2.19	0.98	5.01	0.05
X8	−0.71	0.32	4.87	0.05
X9	−0.37	0.16	4.96	0.05
X10	0.52	0.16	11.08	0.01
X11	0.60	0.19	9.51	0.01

X1–X2 indicates the increment of total starch content of grains 0–23 days and 23–47 days after anthesis, in turn; X3–X5 indicates the variation in the crude protein content of grains 0–23 days, 23–47 days, and 47–75 days after anthesis; X6–X8 indicates the variation in the crude fat content of grains 0–23 days, 23–47 days, and 47–75 days after anthesis; and X9–X11 indicates the variation in the total soluble sugar content of grains 0–23 days, 23–47 days, and 47–75 days after anthesis. The same below.

**Table 2:** Path analysis of grain nutritional quality components.

Index	Correlation Coefficient	Direct Path Coefficient	Coupling Diameter Factor									
			X1-Y	X2-Y	X5-Y	X6-Y	X7-Y	X8-Y	X9-Y	X10-Y	X11-Y	Sum
X1	0.128	−0.603		0.454	−0.012	0.311	−0.151	−0.016	−0.496	0.417	0.224	0.731
X2	−0.846 **	−0.909	0.301		−0.028	−0.148	0.168	−0.004	0.235	−0.301	−0.159	0.063
X5	0.490 *	0.166	0.042	0.155		0.121	−0.046	−0.011	−0.074	0.312	−0.175	0.325
X6	−0.168	−0.610	0.308	−0.220	−0.033		0.342	0.033	0.222	−0.220	0.010	0.442
X7	0.349	−0.390	−0.233	0.392	0.019	0.535		−0.009	−0.147	0.101	0.080	0.739
X8	−0.109	−0.125	−0.078	−0.032	0.015	0.161	−0.027		−0.057	0.140	−0.105	0.017
X9	0.196	−0.603	−0.497	0.355	0.020	0.225	−0.095	−0.012		0.619	0.183	0.799
X10	0.388	0.836	−0.301	0.328	0.062	0.161	−0.047	−0.021	−0.446		−0.183	−0.448
X11	0.027	0.568	−0.238	0.255	−0.051	−0.011	−0.055	0.023	−0.195	−0.269		−0.541

Y indicates the total starch increment of the grain during the period of 47–75 days after anthesis; “*” means significant at *p* < 0.05 and “**” significant at *p* < 0.01.

**Table 3:** Maize cultivars used in this study.

Genotypes Name	Pedigree	Year of Release/Use	Maturity Group
Zhongdan2	Mo17 × Zi330	1975	mid-late maturity
Danyu13	Mo17 ×E28	1986	mid-late maturity
Yedan13	Ye478 × Dan340	1992	mid-late maturity
Xianyu335	PH6WC × PH4CV	2004	mid-late maturity
Denghai618	DH392 × 521	2013	mid-late maturity

## Data Availability

The datasets used and/or analyzed during the current study are available from the corresponding author on reasonable request.
